# An Assessment of Ammonia Emissions from Dairy Facilities in Pennsylvania

**DOI:** 10.1100/tsw.2001.277

**Published:** 2001-10-26

**Authors:** James D. Ferguson, Zhengxia Dou, Charles F. Ramberg

**Affiliations:** University of Pennsylvania, School of Veterinary Medicine, Center for Animal Health and Productivity, Kennett Square 19348, USA

**Keywords:** dairy cattle, ammonia, feed use

## Abstract

A survey of 715 Holstein dairy farms in Pennsylvania was used to construct demographics for the average Holstein dairy farm. The average Holstein dairy farm was composed of 69 lactating cows; 11 nonlactating, pregnant cows; 44 heifers; and 18 calves. Milk production averaged 27.3 kg (60.0 lb). Crop area averaged 73.6 ha. Milk production, crop area and type, average county yields, and herd animal groups were used to construct a typical feeding program for these farms. Typical rations were constructed for six feeding groups (three milk production groups, one nonlactating group, two heifer groups) to meet milk production, pregnancy, and growth requirements. Rations were constructed based on three forage qualities (excellent, average, and poor) typically observed on Pennsylvania dairy farms. Data for animal description (milk production, body weight, growth, and pregnancy status) and ration components and amounts consumed for each animal group were input into the excretion model of the Dairy Nutrient Planner computer program (DNP). Excretion of fecal N and dry matter (DM), urinary N, and total P and K were produced for each animal group and used to assess potential volatile losses of N. Work at the Marshak Dairy, New Bolton Center, indicates the majority of urinary N is rapidly lost as ammonia from dairy facilities. Based on this observation, the losses of N as ammonia were estimated to be 4.63, 4.62, and 4.28 tonne/year for the farm with excellent, average, and poor quality forages, respectively. Volatile losses of N may be reduced most by controlling levels of urea in urine. Urinary N may be reduced through dietary manipulation of protein and carbohydrate sources. Conversion of urea to ammonia may be reduced by altering the pH of barn floors and gutters. Entrapment of ammonia may be accomplished by acidification of manure slurry. Atmospheric ammonia contributes to acid rain, eutrophication of estuaries and lakes, and particulate air pollution. Reduction of ammonia emissions from dairy barns can significantly reduce atmospheric pollution and improve air and water quality.

## INTRODUCTION

Excess N in groundwater, lakes, streams, and estuaries is a significant environmental problem[1]. Excess N loss to the environment can result in eutrophication of estuaries and streams, acidification of nonagricultural soils, deterioration of soil and groundwater quality, and damage to forests and natural ecosystems[1,2]. Nitrate contamination in water as a result of agricultural activity is a problem in regions of the country[1]. Animal waste is a significant contributor to environmental loss of N

through volatilization of ammonia-N, denitrification processes in soil that has received continuous manure application, runoff of surface-applied manure, and/or leaching of N below root zones[2]. Highest concentrations of nitrate-N are found in ground-, surface, and well waters in counties with the highest agriculture activity, especially dairy farms and associated problems with manure disposal. Volatilization of ammonia from animal systems is of increasing concern due to air particulate formation and acidification of rain and soil[3,4].

N in fresh manure is found in three fractions: (1) inorganic salts (ammonia), (2) unstable organic compounds (urea), and (3) stable organic compounds (proteins)[2,5,6]. Urea is rapidly converted to ammonia from urine lying on barn floors and in collection systems[7,8,9]. Sources of stable organic compounds chiefly are undigested feed proteins and endogenous protein in feces[2,4], whereas urea is mainly derived from urine as a result of metabolism of protein[10,11]. Ammonia N in manure is readily available for plant uptake[2], but is also susceptible to volatilization and runoff[2,4,5,9]. Ammonia and urea, due to rapid breakdown to ammonia, are the most labile forms of N in manure, with the most potential to cause volatile losses from animal housing or when manure is applied to soils.

In animal production systems, N is supplied primarily as dietary protein[11,12,13]. Dietary protein is digested into constituent amino acids, which after absorption are used for tissue metabolism. Waste N from indigestible protein and tissue catabolism of amino acids is excreted as protein in feces and as urea in urine[10]. Typically, only 10 to 45% of dietary N is captured in milk or meat[10]. Thus, 55 to 90% of feed N is passed through to manure. Urinary losses are usually higher than fecal losses of N, due to inefficient utilization of N in the rumen and at tissue level[11]. Ration formulation strategy, feed quality, animal grouping, and feed delivery will influence the efficiency of transfer of N from feed to product[10,11,14].

There is a limited amount of data characterizing ammonia losses from dairy barns. Muck characterized losses of ammonia from free stall barns in New York[9]. Muck[9] found that 40 to 50% of the total N in manure could be lost as ammonia from free-stall barns. This loss was sensitive to temperature and wind speed[9]. However, even when temperatures were 10°C, all the urinary N could volatilize if left on the barn floor for 24 h. If the temperature was greater than 20°C, then 40 to 50% of total N could volatilize within a 6-hour period. The source of labile N in dairy manure was urea-N excreted in urine. Frequency of alley scrapping was found to influence the loss of urea-N to ammonia. Spreading lime on alleys increased pH and reduced urease activity, reducing the conversion of urea-N to ammonia and reducing volatile losses[15]. In the Netherlands, Voorburg and Kroodsma[4] estimated that 1 kg of N per cow per month was lost as ammonia from housing units.

Dou et al.[16,17] developed computer software, the Dairy Nutrient Planner (DNP), which predicts forms of N in animal waste based on feed inputs. A survey was conducted to collect information on nutrient management practices on dairy farms in south-central Pennsylvania[18]. The objective of this study was to use feeding and cropping information from the farm survey along with the DNP to predict excretion of total N and forms of N in manure for a typical Pennsylvania dairy farm. Volatile losses of ammonia from the housing system were predicted from the excretion of urea-N for an average Pennsylvania dairy farm.

## MATERIALS AND METHODS

The survey targeted nine counties in Pennsylvania, which were in the Susquehanna River Basin, a major tributary of the Chesapeake Bay. The nine counties comprise 240,000 dairy cows, which represents 37% of the total dairy cows in Pennsylvania[19]. Questionnaires were sent to 3790 farms in September 1996 and repeated in January 1997. A total of 994 surveys were returned.

The questionnaire consisted of 48 queries. The questions sought information on relevant management practices and farm demographics involving breed, animal inventories (milk cows, dry cows, heifers, and calves), average production, feeding methods, feeds, crops grown, land area, and manure handling. Some chose not to answer every question. Only farms with Holstein cows and complete responses to all questions were selected for this analysis.

### Ration Construction and Excretion Prediction

Crop area and average yield per unit area for the county of the farm were used to estimate the volume of dry matter (DM) available for feeding for each crop. Storage losses were estimated to be 10% of yield for forages and 3% for grains. Total forage available on the farm was the sum of all crop forages in storage. Of the farms, 68% reported feeding some pasture to lactating cows; this was only considered a forage option for 25% of the year and only for 10% of forage need. The total volume of crop DM was divided by 365 to estimate the amount available per day for feeding to animal groups.

Four physiologic groups were considered on the farm: (1) lactating cows; (2) nonlactating, mature cows (dry cows); (3) immature, nonlactating cows (heifers); and (4) calves on milk. For these calculations, calves on milk were not included in the feeding budget. Heifers were divided into two groups: (1) 6 months of age and 159 kg body weight; and (2) 19 months of age, 409 kg body weight, and 120 days of gestation. Daily DM intake was calculated for each of the physiologic groups using the following formulas[12]:

$$\text{Milk cows DM, kg/day} = 1.193 + 0.007*BWT_l, + 0.107*Milk + 0.0000285*BWT_l*Milk$$
$$\text{Dry cows DM, kg/day} = 0.015*BWT_d,$$
$$\text{Heifers, DM, kg/day} = 0.018*BWT_h,$$

where $BWT_l$ = 591 kg, $BWT_d$ = 636 kg, $BWT_h$ = 159 and 409 kg, and Milk = kg/day, average production for the farm reported in the survey.

The forage proportion of DM was 40 to 60% for lactating cow diets, 70 to 80% for mature, nonlactating cow diets, 20 to 30% for 6-month-old heifer diets, and 70 to 80% for 19-month-old heifer diets. Total forage DM needed per day was calculated as the sum of the number of animals in each physiologic group (milk cows, dry cows, and heifers) multiplied by the forage DM for each ration group. The proportion of forage DM for an individual crop was calculated as DM for the crop in storage divided by the total forage DM in storage multiplied by the forage DM intake per day. Daily DM of each forage crop was estimated for each of the physiologic groups. The requirements for a year were estimated by multiplying daily needs by 365.

Rations were balanced for each physiologic group to meet requirements for maintenance, milk, gestation, and growth[20]. Growth in heifers was set at 0.82 kg/day to have sufficient size for first parturition at 24 months of age. Rations for the milk production group were constructed for early, middle, and late lactation by establishing a production curve for the average milk reported. The lactating cows in the herd were distributed across early, mid, and late lactation to result in an average milk per day equal to the herd average. Rations were weighted by the number of cows and weeks of lactation to calculate a yearly value for forage and grain requirements and manure production. Time units were 9 weeks for early lactation, 12.6 weeks for mid lactation, and 22 weeks for late lactation with a dry period of 60 days. Two feeding groups were considered for heifers: (1) less than 1 year of age (159 kg body weight and not pregnant) and (2) over 1 year of age (409 kg body weight and 4 months pregnant). This provided rations for six groups of cows on the farm.

Rations were formulated using the DNP[16,20]. Ration constraints were established for each of the six physiologic groups. Animal inputs included body weight, level of milk production and composition, age, gestation stage, and tissue gain per day. Body weights were as described. Milk composition was calculated based on data from the Pennsylvania Dairy Herd Improvement Association (PADHIA) production records for Holstein cows based on the level of production reported in the survey data. Age was two lactations for lactating and dry cows, and 6 and 18 months of age for heifers. Gestation status was a function of stage of lactation and age. Environmental factors were set for average temperature and exposure.

Forage as a percent of DM was set to 40 to 60% for lactating cows, 70 to 80% for dry cows and older heifers, and 20 to 30% for younger heifers. Secondly, constraints were established for neutral detergent fiber (NDF), nonfiber carbohydrate (NFC), rumen N balance, net energy balance (NE), and metabolizable protein balance (MP). NE and MP were constrained to be between 0 and 5% positive. For lactating cows, NDF could range from 30 to 35%, NFC from 35 to 42%, and rumen balance of N of zero. For nonlactating cows, NDF could range from 40 to 50%, NFC from 30 to 38%, and rumen balance of N could vary depending on forages fed. Heifer diets were balanced so NE and MP would meet the input rates of gain. NDF and NFC were allowed to vary, as well as rumen balance of N. Rumen N balance was set to be zero in lactating diets to minimize N excretion from excess rumen N.

Feeds selected for inclusion in the ration formulation strategy were those identified as being used on the farm. If feeds were used on the farm but were not grown on the farm, they were purchased. If total amount of forage grown did not meet DM requirements, then the remaining forage was purchased.

Three levels of forage quality were considered for ration construction: excellent, average, and poor (Table 1). Ranges were based on values observed by the Cumberland Valley Analytical Service, Maugansville, MD. Nutrient concentrations of grains grown on the farm (corn, soy, and small grains, primarily barley) and feeds purchased to balance the rations were based on NRC composition tables[12].

Based on final ration composition, the DNP predicted fecal and urinary N excretion on a per-cow-per-day basis, which was

**TABLE 1**
**Yields and Nutrient Content of Forages and Feeds Grown on Farms in Pennsylvania**

| Forage/Feed | Yield Range Tonne/ha | Composition (%DM) | | | | | Mcal/kg Nel |
|---|---|---|---|---|---|---|---|
| | | DM | CP | NDF | P | K | |
| Corn Silage | 35.9 – 44.9 | | | | | | |
| Quality | | | | | | | |
| Low | | 35 | 7.0 | 50 | 0.21 | 0.95 | 1.43 |
| Average | | 35 | 8.0 | 44 | 0.21 | 0.95 | 1.54 |
| Excellent | | 35 | 9.0 | 40 | 0.21 | 0.95 | 1.61 |
| Alfalfa | 6.3 – 8.8 | | | | | | |
| Quality | | | | | | | |
| Low | | 85 | 16.0 | 52 | 0.35 | 2.21 | 1.21 |
| Average | | 85 | 19.0 | 47 | 0.35 | 2.21 | 1.30 |
| Excellent | | 85 | 21.0 | 42 | 0.35 | 2.21 | 1.41 |
| Grass | 4.5 – 5.6 | | | | | | |
| Quality | | | | | | | |
| Low | | 85 | 7.0 | 70 | 0.28 | 1.99 | 1.08 |
| Average | | 85 | 10.0 | 63 | 0.28 | 1.99 | 1.21 |
| Excellent | | 85 | 15.0 | 55 | 0.28 | 1.99 | 1.41 |
| Corn grain | 7.0 – 8.9 | 90 | 10.0 | 28 | 0.14 | 0.53 | 1.91 |
| Soybeans | 2.6 – 3.1 | 90 | 41.8 | 13 | 0.65 | 1.82 | 2.11 |
| Small grains | 2.6 – 3.1 | 90 | 12.8 | 19 | 0.38 | 0.47 | 1.94 |

*Note:* Data based on forage and feed analysis by Cumberland Valley Analytical Services, Maugansville, MD 21767.

aggregated for 365 days to estimate a yearly production. Total excretion of N was calculated on an animal-unit basis. Ammonia losses were estimated from urea-N.

## RESULTS AND DISCUSSION

A total of 715 farms met the inclusion criteria. The average farm had 69 lactating cows, 11 dry cows, 44 heifers, and 18 calves. Mean milk production was 27.3 kg. Mean reported crop area was 73.6 ha, of which 28.1% was corn silage, 24.9% was legume forage, 28.1% was corn grain, 7.0% was soybeans, 5.9% was grass forage, and 11.2% was pasture.

The primary forages used on farms were corn silage and legume/legume-mixed forage (Table 2). Corn silage was fed to 95% of lactating cows, 86.2% of dry cows, and 82.3% of heifers. Legume forages were fed to 99% of lactating cows, 54% of dry cows, and 74% of heifers (Table 2). Grass forage was reported fed on 21 to 28% of farm diets, depending on animal group. Pasture was used on 69 to 75% of farms. Corn, dry or as high moisture, was the primary concentrate. Soybeans and soybean meal were the primary protein supplements. Of the farms, 35% used a mixed protein supplement from a feed dealer. By-products were not used on a high proportion of farms as direct products. These were probably utilized as part of a dairy feed or protein supplement supplied in a blended product from a feed dealer. The reported percents in Table 2, along with crop area and yield, provided the background to construct rations for each feeding group.

Our approach to estimating forage intake based on percent of land area committed to crop and average yield for the crop appeared reasonable, as 39% of farms reported purchasing forage and we estimated that 36% of farms would need to purchase

**TABLE 2**
**Fraction of Farms Reporting Feeds Fed to Selected Animal Groups on Holstein Dairy Herds (n = 715)**

| Feed | Lactating Cows | Dry Cows | Heifers |
|---|---|---|---|
| Forages | | | |
| Corn silage | 0.950 | 0.862 | 0.823 |
| Legume/Mix forage | 0.990 | 0.540 | 0.743 |
| Grass forage | 0.283 | 0.212 | 0.260 |
| Pasture | 0.687 | 0.748 | 0.715 |
| Grains/concentrates | | | |
| High-moisture corn | 0.519 | 0.284 | 0.252 |
| Dry corn | 0.575 | 0.542 | 0.573 |
| High-moisture small grains | 0.031 | 0.014 | 0.015 |
| Dry small grains | 0.363 | 0.307 | 0.348 |
| Dairy feed | 0.172 | 0.137 | 0.120 |
| Protein supplements | | | |
| Whole soybeans | 0.406 | 0.084 | 0.077 |
| Soybean meal | 0.618 | 0.405 | 0.414 |
| Distiller's grains | 0.300 | 0.093 | 0.075 |
| Whole cottonseeds | 0.334 | 0.034 | 0.023 |
| Corn gluten meal | 0.086 | 0.018 | 0.020 |
| Blood meal | 0.065 | 0.009 | 0.007 |
| Fish meal | 0.049 | 0.003 | 0.004 |
| Animal blend | 0.074 | 0.022 | 0.011 |
| Protein feed | 0.350 | 0.119 | 0.132 |
| By-products | | | |
| Soyhulls | 0.144 | 0.050 | 0.028 |
| Wet brewer's grains | 0.037 | 0.013 | 0.012 |
| Dry brewer's grains | 0.044 | 0.014 | 0.021 |
| Corn hominy | 0.050 | 0.020 | 0.012 |
| Corn gluten bran | 0.008 | 0.001 | |
| Bakery waste | 0.047 | 0.010 | 0.011 |

From Dou et al.[18].

forage based on our use calculations. Our estimates of grain purchases based on overall farm energy and protein balance were higher than reported. Compared with our estimate of 86%, 70% of farms reported purchasing grain. This may represent that farms growing grain had higher than average yields for the county.

An example of farm use of forages and feeds is presented in Table 3 for the average forage quality. The three lactating cow groups and two heifer groups were combined to provide an average daily utilization. The approach to ration balancing was to minimize the number of feeds selected, but provide sufficient energy and protein to support each physiologic group to meet production expectations with no body weight loss and no excessive weight gain. Corn silage represented 59% of the forage used in the ration. Based on the farm survey data, 61% of the forage DM grown on the farm was from corn silage. This was computed based on average county yields for corn silage and crop area reported for corn silage across the farms in the survey. Thus,

**TABLE 3**
**Mean Intake and Excretion of Dry Matter for Ration Ingredients Per Animal Per Day for Three Main Feeding Groups for Average Forage Composition**

| Item | Lactating Cows | Nonlactating Cows | Heifers |
|---|---|---|---|
| Forages, kg/day | | | |
| Corn silage | 6.50 | 4.95 | 2.40 |
| Legume forage | 3.55 | 1.14 | 1.20 |
| Grass forage | 0.64 | 1.82 | 1.20 |
| Grains, kg/day | | | |
| Corn meal | 3.22 | 0.91 | 1.82 |
| Soybeans | 0.44 | | |
| Soybean meal | 1.11 | 0.68 | 0.70 |
| Soyhulls | 0.10 | | |
| Protein supplement | 0.50 | | 0.04 |
| Mineral/vitamin, kg/day | 0.23 | 0.05 | 0.05 |
| Nutrient content | | | |
| CP, % DM | 16.0 | 13.7 | 15.4 |
| P, % DM | 0.34 | 0.30 | 0.37 |
| K, % DM | 2.05 | 2.64 | 3.09 |
| Intake, g/day | | | |
| DM | 16260.8 | 9534.0 | 7350.7 |
| N | 416.6 | 209.0 | 180,8 |
| P | 56.2 | 28.6 | 26.7 |
| K | 334.5 | 251.2 | 223.1 |
| Excretion, g/day | | | |
| Fecal OM | 4425.3 | 2607.5 | 1883.5 |
| Fecal N | 184.5 | 104.6 | 82.8 |
| Urinary N | 127.2 | 102.5 | 72.2 |
| P | 52.9 | 34.7 | 34.3 |
| K | 299.9 | 246.8 | 221.9 |

*Note:* Four physiologic groups were considered: (1) lactating cows (BWT = 591 kg, average milk production 27.3 kg), (2) nonlactating cows (dry cows, BWT = 636 kg), (3) heifers (age = 6 months, BWT = 159 kg), and (4) pregnant heifers (age = 19 months, BWT = 409 kg). Rations were balanced to meet requirements for maintenance, gestation, and production[20]. Growth in heifers was 0.82 kg/day. Rations for lactating cows were distributed across early (9 weeks), middle (12.6 weeks), and late (22 weeks) lactation based on herd average milk yield. Lactating rations were weighted by the number of cows and weeks of lactation to calculate a daily value for forage and grain intake and manure production. Dry period was 60 days. The table presents the mean daily values for lactating, dry, and immature animals.

forage use in the diet was calculated based on commitment to forage crop area and production, as seen above for corn silage use in the ration.

Since farms feed legumes and mixed legumes as haylage or hay, we chose to represent legume feeds as hay in rations, as county yield data was only expressed in hay units. Legume and legume-mixed forages were 25% of the forage DM in the ration (Table 3), whereas farms raised 33% of forage DM from legumes and mixed forages. The remainder of forage DM used in the ration was from grass forages, which was 16% of the total ration forage DM. Based on the farm survey data, farms raised 6% of forage DM as grass. Thus, our estimated ration use was higher than that estimated from the crop area data. However, since many farms reported using pasture (Table 2), we felt that increasing the grass proportion to 16% of ration DM would better account for pasture use. Therefore, although the legume forage use in the ration was lower than indicated from the survey calculation based on crop area, when pasture use was factored in to forage use, legume use declines and grass use increases. Therefore, we feel the forage amounts in Table 3 provide a reasonable approach for ration construction.

The grain amounts in Table 3 are those needed to supply sufficient energy and protein to support production and maintenance requirements. Corn and soybean meal were the major grain sources, as almost all farms utilized corn as dry or high-moisture grains for all groups of cows, and soybean meal was the most common protein supplement reported in the survey (Table 2). A high proportion of farms used a dairy feed or protein supplement from a feed dealer which would have included these grain sources and by-products of animal, ethanol, and milling industries, particularly distiller's dried grains, soy hulls, or wheat midds. We chose to allow soy hulls and distiller's dried grains to be selected as representative of the use of these mixes. A high proportion of farms reported growing and feeding whole soybean seeds, therefore these were also offered in the rations. A small proportion of farms reported using animal-marine blends. These were available in the solutions, but we were not forced in solutions to the feeding groups.

Inputs of N in feed were highest for lactating cows, 417 g/day compared to 209 g/day for dry cows and 181 g/day for heifers. This was expected as higher protein amounts are needed to support milk production. Fecal N and urinary N were highest for lactating cows, 185 and 127 g/day, respectively, compared to the dry cows and heifers. Output of N in milk was estimated based on the average milk production, 27.3 kg, times the average crude protein content at that level of production in Pennsylvania, 3.2%. N output in milk was calculated to be 138 g/day (0.032*27.3/6.33*1000). It was estimated that 21 and 30 g of N were retained per day in the younger and older heifers, respectively. Live weight gain was input at 1.8 kg/day. This represented 7.2 to 10.5% of the live weight gain was protein.

Based on work from Muck[7] and from observations at the Marshak Dairy at the University of Pennsylvania, all urinary N is susceptible to loss as ammonia. Once excreted, urea is rapidly broken down to ammonia by bacterial urease activity in animal waste. Concrete of alleyways is impregnated with urease and even recently scrapped floors have significant activity[4]. As urine spreads over the floor surface, it is subject to urease activity and the contact surface area is high. Therefore, urea conversion to ammonia is rapid. We recover little to no urea from floor surfaces on our dairy alleyways (personal observation). It is reasonable to consider all urinary N subject to volatile loss as ammonia. Muck and Richards[9] found that most of the N lost from manure on barn floors was primarily from the conversion of urea to ammonia.

Fecal N is primarily in organic forms of indigestible protein and bacterial cell wall material and is more slowly degraded to ammonia than urea. Hot weather can enhance breakdown on barn floors to ammonia, but the magnitude of production is small compared to that from urea. During collection in manure pits, organic N can be converted to ammonia. Crust formation on pit surfaces can reduce volatile losses from storage systems. However, agitation at time of disposal and surface application can result in substantial losses of ammonia formed as a result of fecal N breakdown.

Rations for each forage quality group are presented in Table 4 for total feed use per year. Excretion data is presented for fecal DM, fecal N, and urinary N. If it is assumed that all urinary N is volatilized from the dairy barn, then 4.2 metric tons of N are lost as ammonia each year on the average Pennsylvania dairy farm. For the average Pennsylvania dairy herd of 136 animal units (AU, 1000 lb of body weight), this would represent 30.9 kg of N lost per year per AU. Voorburg and Kroodsma[4] reported a value of about 1 kg N per cow per month lost from Dutch housing systems as ammonia. This would calculate to 12 kg of N per year per cow. The estimate in this study is significantly higher than the Dutch estimate, which may be due to higher protein diets fed in the U.S. Higher protein diets are fed in the U.S. because milk production is higher than in the average Dutch condition. In addition, dry matter intake is higher in association with the higher milk production. Muck[7] found that 40 to 50% of total Kejhldahl N was lost from manure when barn temperatures were above 10$^{o}$C. In this study, urinary N represented 39% of excreted N. If all this N was volatilized, projected values of volatile N would be consistent with those of Muck[7].

Forage quality reduced corn grain fed, decreased total amount of soybean seeds and soybean meal that was needed in rations, and reduced total intake N (Table 4) to a slight degree. Fecal excretion was lower on the higher quality forage; however, urinary excretion did not vary to a great extent across forage quality. Efficiency of conversion of intake N to milk was higher on the excellent forage situation, but not dramatically improved over the average- and poor-quality situations. Milk production was held constant in this analysis, which may not be the actual case as milk production may increase with improved forage quality.

Loss of urea to ammonia may be prevented by capturing the urine separately into a sealed structure or a deep structure, by acidifying urine to convert ammonia to ammonium ion to reduce volatilization, or by alkalinizing floor surface with lime to denature urease[4,5,15]. Ammonia losses could be reduced from a free stall barn by frequent scrapping of manure into a lagoon[8]. If manure is captured under a barn floor in an open slatted system, total losses of ammonia are similar to a solid alley system[4]. Thus, collection of urine and feces needs to be capped in some fashion. Rubber membranes, straw, or dried manure material provide sufficient impedance to reduce volatile losses, but then manure must be moved to the structure in a timely and efficient fashion. In Pennsylvania, the majority of farms are tie stall barns with drop gutters located behind cows to capture manure until

**TABLE 4**
**Total Feed Use and Excretion Per Year (Tonne) for Different Forage Quality**

| | | Forage Quality | |
|---|---|---|---|
| **Feed** | **Excellent** | **Average** | **Poor** |
| Corn silage | 222.0 | 222.3 | 222.3 |
| Legume forage | 99.3 | 113.2 | 113.2 |
| Grass forage | 42.6 | 42.6 | 42.6 |
| Corn grain | 120.9 | 121.8 | 148.7 |
| Soybean seeds | 7.5 | 11.0 | 13.9 |
| Soybean meal | 31.9 | 42.4 | 50.2 |
| Distiller's grains | 12.6 | 21.9 | 3.4 |
| Blood meal | 0.3 | | 1.1 |
| Whole cottonseeds | 7.4 | | 3.3 |
| Soyhulls | 34.8 | 5.1 | |
| Intake N | 14.06 | 14.90 | 14.94 |
| Excretion | | | |
| Fecal DM | 144.5 | 157.7 | 172.2 |
| Fecal N | 6.5 | 6.7 | 6.6 |
| Urinary N | 4.2 | 4.8 | 4.9 |
| Output Milk | | | |
| N | 3.47 | 3.47 | 3.47 |
| Efficiency (output/input) | | | |
| N | 0.247 | 0.233 | 0.233 |

*Note:* Four physiologic groups were considered: (1) lactating cows (BWT = 591 kg, average milk production 27.3 kg), (2) nonlactating cows (dry cows, BWT = 636 kg), (3) heifers (age = 6 months, BWT = 159 kg), and (4) pregnant heifers (age = 19 months, BWT = 409 kg). Rations were balanced to meet requirements for maintenance, gestation, and production[20]. Growth in heifers was 0.82 kg/day. Rations for lactating cows were distributed across early (9 weeks), middle (12.6 weeks), and late (22 weeks) lactation based on herd average milk yield. Lactating rations were weighted by the number of cows and weeks of lactation to calculate a daily value for forage and grain intake and manure production. Dry period was 60 days. Daily values were multiplied by 365 to calculate yearly values. The table presents the yearly values for lactating, dry, and immature animals. Excretion data based on the model of Dou et al.[16] Rations were formulated for excellent, average, and poor quality forages based on values presented in Table 1.

removed from the barn. Gutters vary in depth between barns with some being 0.45 to 0.80 m in depth. The deeper collection would reduce ammonia loss compared to a flat floor surface. However, when the material is removed from the barn and spread on fields, the trapped ammonia would be susceptible to volatile losses at this time. Amendment of alley floors and gutter surfaces with lime could inhibit urea conversion to ammonia in barn areas, but timely collection would be necessary to capture this benefit. Therefore, on all farm systems there is an incentive to control urinary losses through ration formulation and feeding management.

To significantly control ammonia emissions from dairy housing systems, urinary N must be controlled. This can be achieved through a multiple approach to the problem. Reduction in ammonia emission may be achieved through ration formulation practices which may reduce urinary N and through manure collection practices which inhibit ammonia volatilization. Urinary N can be reduced by maintaining a high efficiency of N utilization in the ration. This entails first balancing rumen provision of N and carbohydrate to reduce loss of N from the rumen as ammonia, which is excreted as urea in urine. Secondly, total protein in the ration should not be oversupplied for milk production. Feeding excess protein increases rumen losses of N and metabolic losses of N from catabolism in body tissues, resulting in an increase in urinary N. Cattle may be grouped to facilitate more efficient protein feeding based on milk production to minimize overfeeding and reduce urinary N excretion. Manure management strategies need to be incorporated with feeding strategies to minimize ammonia losses after excretion of urine and feces during the collection, storage, and field application processes.

This paper presents a best and worst case scenario for ammonia losses from a Pennsylvania dairy farm in that rations were formulated to minimize N losses from rumen digestion and metabolism, but all urinary N is considered to be lost as ammonia. Rations were balanced to meet rumen and ruminant tissue requirements with no oversupplementation. Thus, urinary losses of N are minimized. The actual balance of N supply from rations may be higher than the rumen may utilize. The average milk urea value on Pennsylvania dairy farms suggest this may be the case. Volatilization of ammonia from urea may be lower from dairy barns with deep gutters. However, losses of ammonia at collection and field application (scrapped often onto stacks or wagons for spreading daily) would eliminate this benefit. Over 60% of farms surface spread manure daily to fields, which would result in volatilization of ammonia at the time of field application rather than from the dairy facility at time of collection[18].

| | | |
|---|---|---|
| Received: | July | 27, 2001 |
| Revised: | September | 21, 2001 |
| Accepted: | October | 5, 2001 |
| Published: | October | 26, 2001 |
